# Transcriptional analysis of *Pinus sylvestris *roots challenged with the ectomycorrhizal fungus *Laccaria bicolor*

**DOI:** 10.1186/1471-2229-8-19

**Published:** 2008-02-25

**Authors:** Gregory Heller, Aleksandra Adomas, Guosheng Li, Jason Osborne, Len van Zyl, Ron Sederoff, Roger D Finlay, Jan Stenlid, Frederick O Asiegbu

**Affiliations:** 1 Department of Forest Mycology and Pathology, Swedish University of Agriculture, Uppsala, Sweden; 2 Forest Biotechnology Group, North Carolina State University, Raleigh, NC, USA; 3 Department of Statistics, NCSU, Raleigh, NC, USA; 4 Department of Forest Ecology, University of Helsinki, Helsinki, Finland

## Abstract

**Background:**

Symbiotic ectomycorrhizal associations of fungi with forest trees play important and economically significant roles in the nutrition, growth and health of boreal forest trees, as well as in nutrient cycling. The ecology and physiology of ectomycorrhizal associations with *Pinus sp *are very well documented but very little is known about the molecular mechanisms behind these mutualistic interactions with gymnosperms as compared to angiosperms.

**Results:**

Using a micro-array approach, the relative abundance of 2109 EST transcripts during interaction of *Pinus sylvestris *roots with the ectomycorrhizal fungus was profiled. The results reveal significant differential expression of a total of 236 ESTs, 96 transcripts differentially abundant after 1 day of physical contact with the fungus, 134 transcripts after 5 days and only 6 after 15 days at early stages of mantle formation on emerging lateral roots. A subset of cell wall modification and stress related genes was further assessed by quantitative reverse transcription PCR at late stages of mycorrhizal development coinciding with Hartig net formation. The results reveal down regulation of gene transcripts involved in general defence mechanism (e.g. antimicrobial peptide) as well as those involved in cell wall modification (e.g. glycine rich protein, xyloglucan endo transglycosylase).

**Conclusion:**

This study constitutes the first attempt to characterize the transcriptome of the plant partner in the *Pinus sylvestris *– *Laccaria bicolor *model system. We identified 236 ESTs which are potentially important for molecular regulation of a functional symbiotic association in conifer host. The results highlight similarities with other studies based on angiosperm model systems, nevertheless some differences were found in the timing and spatial scale of gene regulation during ectomycorrhiza development in gymnosperms. The present study has identified a number of potentially important molecular events responsible for the initiation and regulation of biochemical, physiological and morphological changes during development of a fully functional symbiosis that are relevant for gymnosperm hosts.

## Background

Ectomycorrhizal (ECM) symbiosis is an important feature in the life and health of trees in boreal forests where it plays a major role in nutrient cycling and the functioning of the forest ecosystem. Trees with well developed ectomycorrhizal root tips are more resistant to environmental stresses such as drought and to biotic stresses such as root pathogens [1,2]. The establishment of ectomycorrhizal symbiosis is triggered by signals produced by both partners [3-6]. These signals lead to morphological changes and a complex development of specific structures in both the plant and the fungus. Ectomycorrhizal associations are characterized by the successive development of three structural components: a mantle or sheath of fungal tissue that encloses the root, the intraradical network of hyphae, called the Hartig net, and an extraradical mycelium, which extends into the soil surrounding the root and is responsible for water and nutrient uptake. The Hartig net is the site of metabolite exchange between fungal and root cells[1] The ontogenesis of a functional ECM symbiosis requires finely regulated cross talk in time and space between plant and fungal genetic programs involved in sensing the environment, and in cell-to-cell communication.

The molecular mechanisms involved in the pre-mycorrhizal stage to the adaptation of the plant roots in 1) the forming mantle, 2) the penetration of invasive hyphae between epidermal cells and 3) the subsequent intercellular growth of the Hartig net are still poorly understood. Several molecular approaches and large-scale gene profiling experiments have led to the identification of an increasing number of symbiosis related (SR) genes in plant model systems such as *Betula pendula*, *Eucalyptus globulus*, *Quercus robur *and *Tilia platyphyllos *[7-12]. These studies have shown that the morphological and physiological changes observed throughout ectomycorrhizal development are concomitant with changes in gene expression in both partners and take place at the onset of physical interaction [9,12]. The number of genes and the amplitude of their expression vary in time but seem to be more important at the very early stages of development, soon after contact is made. In the pre-infection stage, the intracellular signalling in plant cells is triggered by G-proteins, which are responsible for effluxes of anions and cations causing membrane depolarizations, extracellular alkalinization, phosphorylation or dephosphorylation of unknown target proteins and finally the release of reactive oxygen species (ROS) [13,14]. Hydrogen peroxide (H_2_O_2_) has been shown to play a crucial role in the establishment of ectomycorrhizal symbiosis [15-17]. Calcium spiking has been demonstrated to be an important intracellular signal involved in the initiation of plant symbiotic gene programming [18,19] and different calcium binding proteins such as calmodulins and calreticulins have been identified in *Eucalyptus *and *Betula sp. *during this stage [9,12]. Profound cytoskeletal rearrangements and intense vacuolar activity have also been described. Cell walls and extracellular matrices are the first cellular structures that undergo drastic changes.

Several studies show the transcription of genes coding for enzymes such as chitinases and proteases [9,12,20] or the release of peroxidases into the extracellular space [21-23] in early mycorrhizal stages. The presumed function of these enzymes is to modify plant and fungal cell wall structures but also to release or create secondary signal molecules that are the bi-products of their activity. Cell walls are connected to plasma membranes where receptors are anchored. At this stage, a large number of genes related to stress signalling, defence and cell rescue are found to be induced in different systems, notably pathogenesis related (PR) proteins. A typical gene expressed during this defence response has homology to the *Bet V1* allergenic protein [9,12]. Their expression is quickly modulated, probably by the fungal partner. Overcoming these general defence responses is a pre-requisite for the two next stages of ectomycorrhizal development in which the mycelium ensheaths the root during mantle formation. Following mantle formation, the hyphae penetrate between the epidermal and cortical cells, increasing the surface area of contact between the symbionts and enabling exchange of metabolites between the symbiotic partners [19]. This process provokes a defence response with up regulation of stress-related genes at the early stages of the interaction, probably to limit the fungal invasion of root tissues [24].

Many of these studies have been performed exclusively with angiosperm host plant species while very few have been carried out using gymnosperm hosts, in particular conifers. Angiosperms and gymnosperms are thought to have separated from each other more than 130–90 million years ago [25]. Some of the angiosperm tree representatives (e.g. poplar) are capable of forming both arbuscular and ectomycorrhizal symbioses whereas conifers are exclusively ectomycorrhizal.

The ecology and physiology of ectomycorrhizal symbiosis in *Pinus spp *are very well documented but basic knowledge about how this symbiosis is regulated at the molecular level is still lacking. Only one paper, which focused mainly on the fungal partner *Laccaria bicolor*, has documented large scale profiling of the molecular events during ECM establishment with a gymnosperm as plant host [26].

In this paper, we present a micro-array study focused on the plant partner in the *Pinus sylvestris *– *Laccaria bicolor *system. The primary objective was to explore the molecular basis for recognition and response mechanisms of conifer roots (*Pinus sylvestris *– Scots pine) following challenge with the symbiotic fungus *Laccaria bicolor*. The analysis of expression of over 2000 expressed sequence tags (ESTs) was performed at three time points: 1, 5 and 15 days post inoculation, which led to the identification of several symbiosis related genes. The regulation of a subset of selected genes was further studied by quantitative reverse transcription PCR (qRT-PCR) at a later time point (30 d.p.i.) when full mycorrhizal colonisation was achieved. The results are discussed in comparison to other published studies in angiosperm model systems.

## Results

### Microscopy

**Figure 1:**
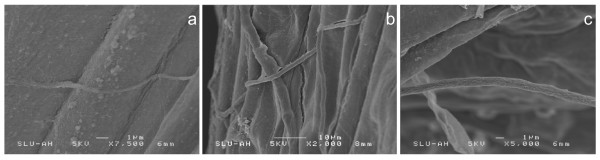
**Scanning Electron Microscopy of the *P. sylvestris *– *L. bicolor system***. SEM pictures of *P. sylvestris *roots interacting with the ectomycorrhizal fungus *Laccaria bicolor *after 1 day (a), 5 days (b) and 15 days (c) post inoculation.

**Figure 2:**
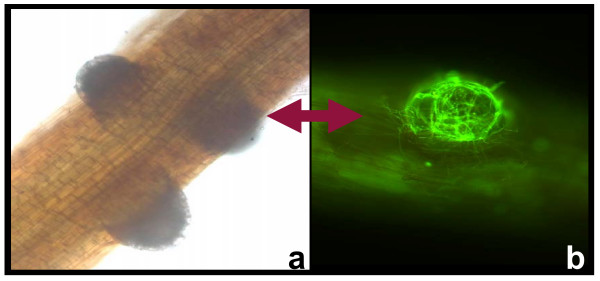
**Light and fluorescent microscopy picture of *P. sylvestris *emerging lateral root**. a) Light microscopy picture of emerging lateral root from *P. sylvestris *at 15 d.p.i. under normal light. b) Fluorescent microscopy picture of the same lateral root stained with acridine orange dye under UV light. *L. bicolor *mycelium is shown as dense and active around this new lateral root.

Active adhesion of the homogenized *L. bicolor *hyphae to the Scots pine roots was visible within a few hours post inoculation. At 24 hours post inoculation the adhering hyphae (Fig. 1a) do not show any growth, but at 5 and 15 days p.i. active growth of the hyphae (Fig. 1b–c) was observed, indicating recovery from homogenization. Despite active growth of the hyphae and increasing physical contact of the hyphae with the pine root, no statistically significant differences in numbers of living cells were detected between the control and mycorrhizal roots at 5 or 15 d.p.i. This suggests that the normal physiology and active meristematic cell division was not perturbed due to the presence of the ectomycorrhizal fungus. At 15 d.p.i. emergence of several lateral roots was observed, accompanied by extensive colonization by the ectomycorrhizal fungus (Fig. 2a–b).

### Analysis of the micro-array data

Statistical analysis of the micro-array data using the 2-mixed model led to a combined total of 236 EST transcripts considered to be differentially abundant with a fold change of at least 1.4 or -1.4 at 1, 5 and 15 days (see Additional file  1). A total of 52, 109, and 5 ESTs had increased transcript levels at 1, 5 and 15 days p.i. whereas 44, 25 and 1 ESTs had a decreased transcript level at same time points. Among transcripts with increased fold changes (>1.4), two (NXSI_036_H01-protein signal transducer and NXCI_005_G03-purple acid phosphatase) were common for 1 and 5 days whereas for decreased transcript levels (-1.4), three were common for 1 and 5 days (37E10-gibberillin regulated protein). Three of these ESTs have no known function.

### General patterns of gene regulation during the early stages of ectomycorrhizal development

**Figure 3:**
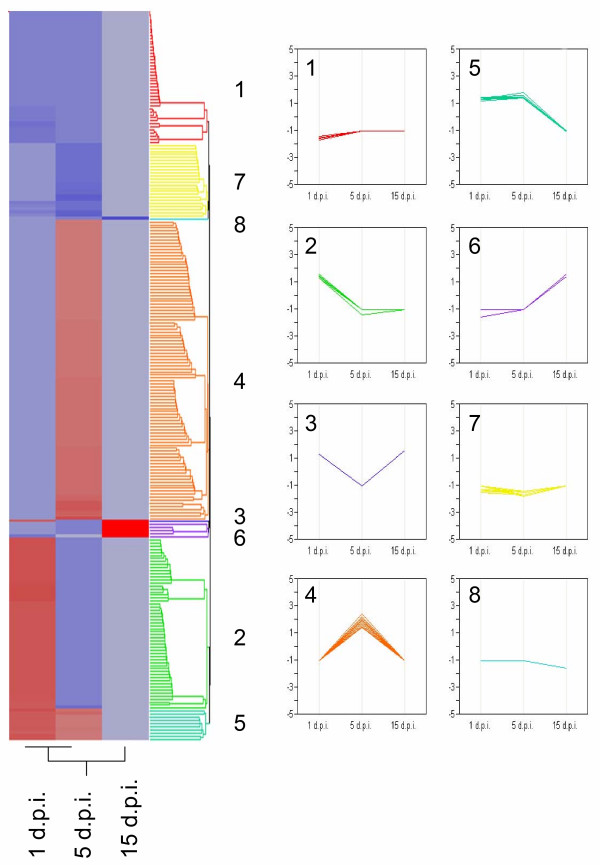
**Hierarchical clustering of micro-array results**. Hierarchical clustering of 236 transcripts from *P. sylvestris *expressed in the roots in response to the presence of the ectomycorrhizal fungus *L. bicolor*. Each row illustrates the expression profile of each of the significantly expressed genes (identified by mixed model analysis). Light red to deep red indicates increasing levels of expression and light blue to deep blue represents decreasing levels of expression). These ESTs have been divided into 8 regulatory patterns, as indicated by the numbers 1–8.

The 236 genes that were differentially expressed were grouped according to a hierarchical cluster analysis (Fig. 3). Within the clusters, eight different expression patterns were identified. Cluster 1 contains genes that were down regulated at the initial phase of interaction but which showed no significant differential expression at the other time points (5 and 15 days). The genes identified within this cluster include those encoding secreted peroxidase and associated with disease resistance (CC-NBS-LRR, TIR-NBS-LRR). The second cluster contains genes significantly induced at 1 d.p.i. but with no differential expression after 5 and 15 days (e.g. H+ transporting ATPase, PREG-like protein, laccases). In addition, several ESTs coding for cell rescue and defence responses are also present in this cluster as well as an MLO protein homologue which plays an important role in recognition by the plant during contact with pathogenic fungi.

Cluster 4 contains the most ESTs and represents genes that are up regulated at 5 d.p.i. but have no differential expression at 1 or 15 d.p.i on the array. Most ESTs involved in protein fate and processing are well represented here (e.g. ubiquitin ligase, GST, chaperone proteins, COP9 signalosome complex subunit). Cluster 5 contains genes that were up regulated at 1 and 5 days but not at 15 days (e.g. purple acid phosphatase, protein binding/signal transducer). The genes identified in cluster 6 have no homology to any known protein in GenBank. The genes in this cluster were all induced at 15 d.p.i. and have no differential expression at the other time points. On the other hand, cluster 7 contains ESTs that are repressed at 1 and 5 d.pi. and have no differential expression at 15 days (e.g. gibberellin regulated protein, NBS/LRR protein). Clusters 3 and 8 are single ESTs and are, respectively, a glycine rich protein homolog induced at 1 and 15 d.p.i. and a pathogenesis related protein PR10 repressed at 15 d.p.i.

### Functional classification of differentially expressed genes

The 236 differentially expressed genes were functionally classified; 21% of the ESTs encode proteins involved in plant metabolism, 11% encode proteins involved in cell rescue and defence, 7% were associated with protein fate (folding, modification and destruction), 7% with energy, 5% with cellular communication and signal transduction and 4% with cellular transport and transport mechanisms (see Additional files 1 and 2). A large proportion of the ESTs (21%) were found to have very weak or no homology to any known gene in the databases.

### 1 d.p.i

The vast majority of ESTs that were differentially expressed were documented within 1 to 5 days post inoculation. About 13% of these ESTs encode proteins that are involved in cell rescue and defence. Genes encoding an MLO protein homolog and a laccase were identified in this group. The role of MLO protein in plant recognition and response to fungal invasion has been highlighted earlier. Several disease resistance protein homolog transcripts including a TIR/p loop/LRR were up regulated at 1 d.p.i. in the presence of the fungus. One EST encoding a peroxidase (EC 1.11.1.7) was down regulated.

For the ESTs related to cell communication and signal transduction, a CC-NBS-LRR resistance like protein transcript was found to be decreased together with a serine threonine protein kinase transcript. A calreticulin, a NBS-LRR disease resistance protein transcript, a 14-3-3 protein transcript and several transcripts of proteins with binding domains or signal transduction functions were found to be up regulated at this time point. A transcript encoding a member of the RAN binding protein family, necessary for nuclear translocation of molecules, was down regulated. Four ESTs related to the transcription process were also found at this stage of the interaction between Scots pine and the ECM fungus. The transcript for a phosphatase regulation gene (coding for the PREG like protein) involved in the regulation of enzymes important for nutrient acquisition was significantly up regulated.

Most of the transcripts for ESTs belonging to the functional category cellular transport and transport mechanisms were found to be down regulated. These include a phosphate transporter and a membrane intrinsic protein (porin MIP). Two other ESTs with homology to genes involved in vacuolar functions and proton transfer or transportation were identified within this group. In contrast, a transcript for an aquaporin was significantly increased in contrast to the findings of other authors [8]. A large proportion of the transcripts differentially abundant at 1 d.p.i. belong to the functional category plant metabolism (18%) and in particular amino acid biosynthesis. Transcripts in this category include arginine decarboxylase and adenosylmethionine decarboxylase (E.C. 4.1.1.50) as well as adenosyl methionine decarboxylase, which were all significantly down regulated in abundance. Interestingly, an inorganic phosphatase was also found to be down regulated. Four transcripts for ESTs belonging to the functional group Regulation of interaction with environment shared homology to genes associated with the phytohormone auxin were all repressed at this stage. Furthermore a significant proportion of the transcripts for ESTs (16%) have no hit or homology to any protein in GenBank.

### 5 d.p.i

The functional category that contains the most ESTs at 5 d.p.i. was metabolism. Several transcripts encoded proteins that were homologous to enzymes involved in cell wall modification (e.g. xyloglucan endo transglycosylase and a beta-xylosidase). Both transcripts were increased and, together with clavata1 receptor kinase, all are known to be important components facilitating plant development during cell wall remodeling associated with lateral root formation. Furthermore the transcripts for alpha pinene synthase were found to be down regulated. This enzyme is involved in the production of alpha pinene, a known antifungal compound.

About 13% of the transcripts for ESTs identified at this time point belong to the cell rescue and defence functional category (e.g. Avr9 elicitor response protein like, TIR/NBS/LRR disease resistance protein, metallothionein like protein, thaumatine like protein PR5). The next predominant functional category concerns Cellular communication and signal transduction mechanisms (10%). Several genes involved in signal perception and transduction have been highlighted at this stage of interaction. A protein kinase receptor was found to be up regulated together with a TIR/P Loop/LRR homolog and a protein binding/signal transducer (up regulated at 1 and 5 d.p.i.). Several ESTs related to protein fate were differentially expressed at 5 d.p.i. representing 10% of the ESTs at this time point. A copper chaperone molecule, a polyubiquitin and a ubiquitin precursor are examples of ESTs found in this functional category.

### 15 d.p.i

At this time point, the micro-array screening identified six transcripts for ESTs to be differentially abundant. Among these ESTs, a transcript for glycine rich protein homolog was found to be up regulated. More interestingly, another gene, homologous to the pathogenesis related protein PR10, was found to be down regulated at 15 days post inoculation. The other ESTs have no known functions.

### qRT-PCR verification

Independent verification of micro-array data was performed using qRT-PCR analyses of fourteen selected genes from specific time points. Analysis of results from both qRT-PCR and cDNA micro-array data, showed that the trends and patterns are completely consistent between the two different methods in estimating fold differences in expression levels from all three time points. As expected the fold values of transcripts detected by qRT-PCR were all higher than those detected by the micro-array, essentially, confirming the micro-array data (Table 1).

**Table 1:** Verification of the array results

	ID	DESCRIPTION	Array	**qPCR**
1 d.p.i.				
	40_A03	membrane intrinsic protein/Porin MIP1	-1,4	**-2,3 **± 1,65
	23_G07	putative auxin induced	-1,4	**-1,7 **± 0,19
	NXCI_055_D02	anthocyanidine synthase	1,4	**3,7 **± 0,38
	NXCI_124_A12	disease resistance protein	-1,4	**-2,7 **± 0,20
	NXNV_134_H10	unknown protein 2	-1,6	**-1,8 **± 0,43
	NXSI_001_G04	unknown protein 3	-1,5	**-2,9 **± 0,30
	03_F07	unknown protein 4	1,5	**3,9 **± 0,22
	NXCI_018_G04	aldehyde dehydrogenase homolog	1,6	**2,5 **± 0,21
5 d.p.i.				
	NXNV_132_G06_F	endoglucanase 1 (ec 3.2.1.4)	1,4	**6,7 **± 1,72
	NXNV_096_C08_F	PR10/BetV1	-1,2	**-5,3 **± 0,10
	NXSI_021_A09	Clavata1	1,4	**5,3 **± 0,76
	NXCI_085_H12_F	tau class glutathione S-transferase	1,6	**7,3 **± 0,99
	02_C09	subtilisin like protease precursor	1,6	**10 **± 0,80
15 d.p.i.				
	NXNV_096_C08_F	PR10/BetV1	-1,6	**-2,5 **± 0,74

Fold changes of selected *P. sylvestris *gene transcripts (± standard deviation) determined by qRT-PCR in root tissues challenged with *L. bicolor *at 1, 5 and 15 d.p.i. as compared to uninoculated control.

### qRT-PCR analysis of gene expression at a later stage of ectomycorrhizal development

To further analyse the expression levels of a subset of selected genes from the array during ectomycorrhizal development, root tissues were harvested from new biological material from a hydroponic system at a later stage of ectomycorrhizal development (30 d.p.i). These results are presented in Table 2. For this analysis, qRT-PCR was performed on 20 selected genes instead of large scale profiling. Two genes found to be down regulated after 1 day, but without differential expression at 5 and 15 d.p.i., were up regulated at 30 d.p.i. (putative auxin induced and MtN21 nodulin like). These genes are known to be involved in plant microbe interactions and are believed to be regulated by the phytohormone auxin. Auxins are also produced by ectomycorrhizal fungi and play an important role in ectomycorrhizal development [27].

**Table 2:** Transcript abundance of a subset of *P. sylvestris *ESTs at 30 d.p.i. determined by qRT-PCR.

ID	DESCRIPTION	Array			**qPCR**
		1 d.p.i.	5 d.p.i	15 d.p.i.	30 d.p.i.
23_G07	putative auxin induced	**-1,4**	-1,0	-1,1	**2,5 **± 0,08
NXPV_043_G04_F	MtN21 nodulin like protein	**-1,6**	-1,1	-1,2	**1,9 **± 1,14
NXSI_021_A09	clavata1	1,2	**1,4**	-1,0	**-2,0 **± 0,05
NXCI_098_F10_F	chalcone flavone isomerase	1,0	1,2	1,2	**-1,5 **± 0,06
NXCI_055_d02	anthocyanidine synthase	**1,4**	-1,0	1,1	**2,0 **± 0,71
NXSI_012_h05	s adenosylmethionine synthase	1,0	-1,0	1,2	**-2,4 **± 0,19
40_A03	membrane intrinsic protein	**-1,4**	-1,3	-1,0	**-1,6 **± 0,07
involved in Stress and Defense reactions					
NXCI_085_H12_F	tau class glutathione S-transferase	-1,2	**1,6**	-1,0	**1,5 **± 0,26
NXNV_096_C08_F	PR10	1,0	-1,2	**-1,6**	**-1,0 **± 0,06
02_C09	subtilisin like protease precursor	1,1	**1,6**	1,1	**-5,6 **± 0,07
NXNV_162_h07	Thioredoxin	-1,2	-1,1	-1,2	**1,1 **± 0,14
NXSI_012_D08_F	Peroxidase	1,0	1,1	1,3	**2,8 **± 0,30
4_G06	antimicrobial peptide	1,2	-1,0	-1,1	**-5,6 **± 0,07
NXSI_064_A03_F	thaumatin	1,2	-1,2	-1,0	**2,1 **± 0,70
involved in Cell Wall Modifications					
NXSI_054_F05	glycine-rich protein	1,3	1,0	**1,6**	**-11,5 **± 0,03
NXNV_132_G06	endoglucanase 1 (ec 3.2.1.4)	1,2	**1,4**	1,0	**-2,5 **± 0,16
02_B03	cinnamoyl alcohol dehydrogenase	1,0	-1,1	1,1	**1,4 **± 0,49
NXCI_066_h04_F	acetoacetyl CoA thiolase	1,1	-1,0	1,0	**2,1 **± 0,32
NXNV_164_H08	xyloglucan endotransglycosylase	1,0	-1,2	1,1	**-2,8 **± 0,15
34_F04	cinnamoyl CoA reductase	1,2	-1,0	1,0	**-3,6 **± 0,22

Fold change (± standard deviation) of a subset of *P. sylvestris *gene transcripts chosen for their functional relevance and used to monitor their transcript abundance at late stages of ectomycorrhizal development (30 d.p.i.).

The membrane intrinsic protein encoding transcript down regulated at 1 d.p.i., was also found to be down regulated at 30 d.pi. The transcript for anthocyanidine synthase involved in flavonoid biosynthesis was significantly induced at 1 d.p.i. on the array but without differential expression at 5 and 15 days. The gene was found to be up regulated again at 30 d.p.i. In contrast, the transcript encoding a chalcone flavone isomerase, another enzyme involved in the production of flavonoid, was not differentially abundant within the first 15 days but was decreased at 30 d.p.i. Several transcripts with homology to either Clavata 1, endoglucanase 1 or to the subtilisin-like precursor were found to be transiently abundant from 1 to 30 d.pi. with up regulation at 5 d.pi. on the array and down regulation at 30 d.pi Glutathione-S-transferase transcript was up regulated at 5 and 30 d.p.i. but not at 1 and 15 d.p.i The gene encoding PR10 protein was up regulated only at 15 d.p.i. but decreased during the subsequent period of active mycorrhiza formation. Similarly the transcripts involved in cell wall formation such as that encoding a glycine rich protein were increased at 15 d.p.i and decreased at 30 d.p.i.

The transcript levels for genes related to stress responses and encoding peroxidase, thaumatin, cinnamoyl alcohol dehydrogenase and acetoacetyl CoA thiolase were also studied. The genes were selected for their potential role in the defence response of pine seedlings against fungal invasion. These genes were all found not to be significantly differentially expressed after 1, 5 and 15 days of interaction with the ECM fungus but showed differential expression at 30 d.p.i. with qRT-PCR. On the other hand, some other defence related genes with homology to cinnamoyl CoA reductase, chalcone flavone isomerase, S-adenosylmethionine synthase and antimicrobial peptide were repressed at 30 d.p.i. corresponding to the period of active mycorrhization or Hartig net formation.

## Discussion

As stated earlier in the introduction, the genetic programmes regulating ECM formation have so far mostly been studied in angiosperms. Both from an economic and an ecological perspective, gymnosperms are also major tree species in boreal forest ecosystems and understanding the molecular basis of mutualistic associations that are beneficial for their growth is an important research priority. In the present study, using an *in vitro *system, we conducted transcript profiling at different stages of mycorrhizal development of *P. sylvestris *with *L. bicolor *involving hyphal associations with both primary and lateral roots.

For the transcript profiling, a heterologous array consisting of 2109 ESTs was used to monitor gene expression at different time points post inoculation. Although the arrays used in this study contain cDNAs from *P. taeda*, we hybridized the arrays with RNA harvested from its close relative *P. sylvestris *roots. The high degree of cross hybridization between *P. taeda *arrays and *P. sylvestris *has earlier been demonstrated by van Zyl [28]. The number of studies using *P. taeda *micro-arrays proved these arrays to be a common and acknowledged tool for assessing differential gene expression in several species belonging to the Pinaceae [29,30].

In this study, 236 transcripts were found to be differentially abundant during the early stages of ectomycorrhizal development. The numbers of differentially abundant transcripts increased during the initial phase of the interaction with a decline at 15 days, coinciding with the first stages of physical contact and lateral root formation respectively. Such periodical and transient changes in gene expression indicate a non-specific nature of host response during the early stages of interaction. The down regulation of several defence and cell wall related genes at late stages of mycorrhization could represent an attempt by the host to accommodate the symbiont. Interestingly, many of the initial changes in the transcript profile during the early stages of interaction represent transcripts involved in metabolism, cell rescue/stress related responses and protein fate. The over representation of transcripts belonging to the functional categories metabolism, cell rescue/stress related responses and protein fate are in line with data reported in other studies using *Betula pendula *[8] or *Eucalyptus globulus *[7,12]. On the other hand, some genes commonly documented in these studies of mycorrhiza development, such as those encoding metallothioneins [12] and chitinases [8,9] were less frequently represented in our system. They represent broad spectrum responses to the presence of the ectomycorrhizal fungus and are believed to be expressed in order to limit invasive growth of the symbiont [24]. Evidence for fatty acid change or transport is a striking feature of the early stages of interactions between *P. sylvestris *and the ectomycorrhizal fungus *Pisolithus tinctorius *[31] but was missing in the present study. These differences may partly reflect the numbers of gene families within these groups that are present on the array used in this study.

In addition, differences between our results and those reported by other authors may be related to the different tree species, the experimental microcosm conditions (including different methods of inoculation) and different temporal programmes required for functional ectomycorrhiza development in the various tissues. For example in the *Betula pendula *– *Paxillus involutus *system, the mantle starts to form within two days and by eight days the Hartig net is already visible in cross sections of the root tip and active mycorrhiza are formed after 21 days [8]. This contrasts with our system where colonization of lateral roots was visible within 15 days but intercellular growth within the cortex was first observed at 30 days. A common feature described in all systems, also documented in our study, is the transient expression of different stress-related genes during the interaction process. The cyclical changes in expression of genes encoding enzymes involved in cell wall modification suggests that they play a significant role in the control of hyphal penetration inside the roots. About twenty ESTs spanning different functional groups were used for further transcript profiling analysis in our system during the later stages of mycorrhiza development. A number of interesting patterns emerged from abundant changes in a number of ESTs encoding proteins involved in cell wall development or cell rescue, defence and stress related functions.

One interesting feature is the regulation pattern of the transcript encoding an antimicrobial peptide. This transcript abundance is increased during initial contact of *L. bicolor *hyphae with pine roots at 1 d.p.i. but decreased at the other time-points. At 30 d.p.i. the antimicrobial peptide transcript abundance was again strongly decreased. The initial increase suggests a non-specific response by host tissue but it is possible that upon recognition of this fungus as beneficial partner the gene is turned off. In pathogenic systems, over expression of AMP has been shown to reduce invasive growth by fungal pathogens [32]. In the present study down regulation of the gene encoding AMP following recognition of the symbiotic fungus *Laccaria bicolor *may be a prerequisite for the penetration of the hyphae and subsequent accommodation of the invading hyphae within the pine root tissues. Other ESTs within the above category code for PR10 and PR5 and have similar profiles on the array as the AMP. They are both slightly increased at 1 d.p.i. and then decreased at 5 and 15 d.p.i. with abundant changes at 30 d.p.i.. PR10, which has homology to the *BetV1* gene family also highlighted in other ECM systems, continued to be decreased at 30 d.p.i In contrast PR5, which is a thaumatin-like protein with anti-fungal properties [33], was found to be increased at 30 d.p.i. which coincides with the period of intercellular hyphae penetration inside epidermal and cortical tissues. It is however difficult to provide an explanation for the differences in regulation pattern of these two PR-protein transcripts, but as documented in other studies the increases in PR5 could be transient. Furthermore, we also documented cyclical regulation of abundance of another stress related transcript, glutathione-S-transferase. The transcript was found to be decreased at 1 d.p.i., increased at 5 d.p.i., then decreased again at 15 d.p.i. and increased at 30 d.p.i In contrast the thioredoxin transcript assessed in this experiment was constantly decreased at 1, 5 and 15 d.p.i. but slightly increased at 30 d.p.i Thioredoxins are involved in response to pathogens and oxidative stresses [34]. Such up and down regulation of several genes belonging to the same functional group underlines the complex nature of the interaction. It is possible that such genes possess dual functions apart from involvement in host defences. It is also most probable that the induced expression in many of these stress or defence related genes are provoked each time the hyphae attempt to enter into new cellular tissues.

Apart from genes with defence related functions, the regulation pattern of transcripts involved in cell wall modification was also interesting. One of the transcripts in this category encodes glycine rich protein (GLP) which was found to be considerably decreased at 30 d.p.i. whereas it was increased at all time points in the array results. GLPs represent the third group of structural protein components for cell walls. They can be exported to neighbouring cells where they contribute to cell wall strengthening [35]. A similar observation was made for another cell wall modification EST (xyloglucan endo transglycosylase (XET)), which was decreased at 30 d.p.i XET may function in modifying cell walls to allow reinforcement of regions under mechanical stress [36]. The concomitant decrease in transcripts encoding GLP and XET suggests a cell wall softening which may be a preparative step for intensive transport mechanisms observed in mature mutualistic associations. Equally interesting is the transcript profiling pattern of genes encoding important enzymes in lignin biosynthesis [37] (cinnamoyl alcohol dehydrogenase (CAD), cinnamoyl CoA reductase (CCR), peroxidase). CCR has been characterized as an effector in defence signalling in rice [38]. The decrease in abundance of its transcript at 30 d.p.i. can be interpreted as a cell wall softening but also an attenuation of a defensive reaction in *P. sylvestris *allowing the presence of fungal hyphae between plant root cells. Peroxidase transcript, unlike CCR, was increased through all the developmental stages assessed. Peroxidase is one of the last enzymes involved in lignin biosynthesis. Peroxidases have also been implicated in plant defence reactions where they play an active role in strengthening plant cell walls at the site of fungal invasion [39,40].

The fold values observed with the micro-array used in this study were generally lower than others reported in the literature. A number of other authors have reported systematic bias in micro-array technology [41]. In the present study, it was necessary, for technical reasons, to use SMART™ PCR to amplify all the RNA samples isolated from *P. sylvestris*. This method is efficient for amplifying RNA exponentially but this non linear amplification could result in a target in which sequence representation is skewed when compared to the original mRNA pool [42,43]. This might have been the case in our study where fold changes were generally lower with the array compared to qRT-PCR. Nevertheless, the stringency and power of the statistical methodology employed for the 2-mixed model analysis [44] allowed us to detect statistically significant changes in transcript abundance with fold changes as low as 1.4. The expression levels of fourteen genes, representing six per cent of the differentially abundant transcripts on the array were verified using qRT-PCR and were found to be completely consistent with the array data. The fold changes, obtained with qRT-PCR, were generally higher compared to array data, as found in other studies [45,46].

Furthermore, the detectable low changes observed in our study with micro-array compared to angiosperm systems could also be due to several other factors. In our earlier study [47], we reported that in gymnosperm pathosystems, host responses are much slower compared to widely studied angiosperm systems. In addition results from the histochemical microscopy studies confirmed that pre-penetration events, mantle and Hartig net formation occurred much later in our system compared to angiosperm systems. Therefore, it is possible that all these together with technical aspects of RNA amplification may have contributed to the artificially low fold changes.

## Conclusion

In summary, the micro-array approach proved to be an adequate technology to study patterns of gene regulation during the development of ectomycorrhizal symbiosis in *Pinus sylvestris*. According to our results, the development of ECM symbiosis in this gymnosperm host appears largely comparable to that in angiosperm hosts. Nevertheless some differences were found in the timing and spatial scale of gene regulation during ectomycorrhiza development in gymnosperms. The present study has identified a number of potentially important molecular events responsible for the initiation and regulation of biochemical, physiological and morphological changes during development of a fully functional symbiosis that are relevant for gymnosperm hosts.

## Methods

### Plant material, fungal inoculum and experimental design

Scots pine (*Pinus sylvestris*) seeds (provenance Eksjö, Sweden) were surface sterilized with 33% hydrogen peroxide (H_2_O_2_) for 15 min, rinsed in several changes of distilled water, sown on 1% water agar Petri plates and left to germinate under a photoperiod of 16 hr light at a temperature of 21°C. The ectomycorrhizal fungal inoculum (*Laccaria bicolor *(Maire) Orton, strain S238 (Crater Lake National Park, Oregon) was obtained after 21 days of growth in liquid Hagem medium [48]. The mycelium was washed with sterile, distilled water and homogenized for 60 seconds in a sterile blender. For the inoculation, the 14 day old Scots pine seedlings were transferred under sterile conditions onto a wet filter paper on a new 1% water agar Petri plate. The seedling roots were inoculated with 1 mL of homogenized mycelium and covered with another wet, sterile filter paper. Control plants were inoculated with 1 mL of sterile distilled water. Each Petri plate was sealed with parafilm and the lower half wrapped with an aluminium foil to protect the roots from light. Prior to sampling the seedlings were then incubated under a photoperiod of 16 hr light at a constant temperature of 21°C. After 1, 5 and 15 days post inoculation (d.p.i.), the roots were frozen in liquid nitrogen, ground and stored at -80°C. Three biological replicates of 100 seedlings were harvested for control and inoculated plants at every time point.

Inoculation experiments for the additional time point, at 30 d.p.i. were slightly different. In this experiment, the two-week-old seedlings were transferred into 15 ml sterile plastic tubes (120 × 17 mm polypropylene tubes, product number 62-554-502, Starstedt, Germany) containing 10 ml of either water (control) or fungal homogenate and incubated under the same growth conditions as in the other experiments. A hole was made through each cap of the 15 ml tube and the root was inserted through it into the solution containing the fungal inoculum. The hole in the cap was sealed with parafilm to prevent microbial contamination at the same time allow gaseous exchange. Aluminium foil was wrapped around the tube to protect the roots from light. Three biological replicates were prepared and the roots were harvested and stored as described above.

### Cell death assay using fluorescence microscopy

The root regions of the main root axis (10 mm) from ten seedlings were harvested at 1, 5 and 15 d.p.i Nuclear staining was carried out as previously described [49]. The roots were first hydrolysed in 3% HCl and 95% ethanol for 5 minutes at room temperature and then washed twice in a phosphate citrate buffer of pH 3.8. Finally the staining was carried out by immersion of the root tips in a phosphate buffered solution containing 0.001% acridine orange for 15 min at room temperature, followed by two washes in citrate phosphate buffer. Only active nuclei within living cells will become fluorescent. The numbers of fluorescent nuclei were counted in a microscope field of view at ×40 magnification and under an excitation filter I_2_: BP 450–490. There were three biological replicates for each treatment.

### Scanning Electron Microscopy

The apical regions (10 mm) of the main root axes of five seedlings were harvested after 1, 5 and 15 d.p.i Fixation was performed in 3% (v/v) glutaraldehyde followed by three washes of 10 min each in a phosphate buffer at room temperature. Subsequent post-fixation was carried out in 1% (w/v) osmium tetroxide for three hours. After four washes in distilled water (4 × 15 min), the roots were dehydrated by successive immersion into ten solutions with an increasing percentage of ethanol (ranging from 10% to 100%, 10 × 10 min), then into a series of ethanol:acetone solutions with an increasing proportion of acetone (3:1, 2:2, 1:3, pure acetone). The samples were finally dried using a Polaron critical point dryer and mounted on stubs. Gold coating was carried out with a Polaron E5000 sputter coater and the samples were observed using a Hitachi S-4500 scanning electron microscopy (SEM) operated at 15 kV.

### Micro-array procedure

The micro-arrays used in this experiment to assess the transcriptional changes of *Pinus sylvestris *roots genes challenged by the ectomycorrhizal fungus *Laccaria bicolor *contained 2,109 ESTs from *Pinus taeda*. The experimental procedures, target preparation and statistical analysis were as previously described [47]. Briefly, the 2109 ESTs (expressed sequence tags) used for micro-array construction were obtained from six cDNA libraries of *P. taeda*. The DNA from each EST was printed onto amino-silane coated slides in four replications for hybridizations performed at 1 d.p.i. and in two replications at 5 and 15 d.p.i. For target preparation, total RNA was isolated from infected and control roots of *P. sylvestris *seedlings. The cDNA was synthesised from the same amount of RNA (1 μg) using SMART™ PCR cDNA synthesis kit (Clontech, USA). The cDNA generated from infected and control roots at each point was reciprocally labelled with Cy3 and Cy5-dUTP (Perkin Elmer, USA) using a Klenow method. Labelling, hybridization, and stringency washes followed the protocol from North Carolina State University. Slides were scanned and raw, non-normalized intensity values were registered using Quantarray software (GSI Lumonics). The experimental design involved comparison of inoculated versus un-inoculated samples at each time point: 1, 5 or 15 d.p.i. Taking into consideration dye-swaps and technical replicates, each sample was hybridized six times and there were a total of 72 data points for each gene on the array at 1 d.p.i. and 36 at 5 and 15 d.p.i.

The statistical significance of changes in transcript abundance was estimated using two successive mixed models as previously described [44,50]. Expression changes of transcripts less than two-fold have been shown to be statistically significant. The genes were considered to be differentially expressed if they met both the following criteria: i) an average fold change of 1.4 in two out of three biological replicates or in all three biological replicates ii) at a p value < 0.01.

The data discussed in this publication have also been deposited at NCBI Gene Expression Omnibus (GEO) [51] and are accessible through GEO platform GPL4039, series accession numbers: GSE5407, GSE5408 and GSE5410.

### qRT-PCR analysis of gene transcription

A subset of genes was used to verify the micro-array results and the expression of an additional subset chosen due to their functional relevance was followed during a late stage of ectomycorrhizal development. 1 μg of total RNA was digested with deoxyribonuclease I (Sigma, Sweden) according to manufacturer's instructions and quantified using Quant-iT RiboGreen RNA Assay Kit (Molecular Probes, Invitrogen, Sweden). The RNA samples were reverse transcribed with M-MLV reverse transcriptase (Invitrogen, Sweden) and as a positive control of the reverse transcription, 5000 copies of kanamycin mRNA (Promega) were added to the reaction mixture. All specific primer pairs were designed using Primer3 [52] with the following characteristics: primer size 20 bp, Tm 60°C and amplicon size from 50 to 150 bp (see Additional file 3). The relative transcript abundance was detected by the ABI Prism 7700 Sequence Detection System (Perkin-Elmer Applied Biosystems, Sweden) using SYBR Green PCR Master Mix (Applied Biosystems) according to manufacturer's recommendations. Transcript levels were calculated using the standard curve method (User Bulletin #2, ABI Prism 7700 Sequence Detection System, Applied Biosystems) made from serial dilutions of known quantity of plasmid (10^6^, 10^5^, 10^4^, 10^3^, 10^2 ^copies/μl). The plasmid DNA concentration was determined using Quant-iT PicoGreen dsDNA Kit (Molecular Probes, Invitrogen, Sweden). The correction for reverse transcription reaction based on kanamycin amplification was included in the calculations. The absolute quantity of the product in each sample was calculated from the standard curves and was normalized against the total amount of RNA as described previously [53].

## Abbreviations

cDNA: clonal deoxyribonucleic acid; ECM: ectomycorrhiza; EST: expressed sequence tag; mRNA: messenger ribonucleic acid; PCR: polymerase chain reaction; qRT-PCR: quantitative reverse transcription polymerase chain reaction.

## Authors' contributions

GH: micro-array study, verification, microscopy, preparation of biological material and qRT-PCR analysis of 30 d.p.i and writing manuscript. FA, AA, GH: micro-array studies at 1,5 and 15 d.p.i. respectively. GL: preparation of biological samples for 15 d.p.i. JO: statistical analysis. LZ, RS: generated cDNA clones and arrays of *Pinus taeda *and drafting manuscript. FA, RF, JS: conceived the study, participated in its design and co-ordination and helped to draft the manuscript. All authors have read and approved the final version of the manuscript.

## Supplementary Material

Additional file 1 Gene transcripts differentially abundant in response to challenge with *L. bicolor *at 1, 5 and 15 d.p.i. Table showing the 236 ESTs from *P. sylvestris *found differentially abundant by the mixed model analysis (cut-off fold change was ≥ 1.4 or ≤ -1.4). All the ESTs were divided into 8 patterns, indicated by the numbers 1–8. The transcript ID can be used to retrieve ESTs sequences from the GeneBank or the database at [54]. Click here for file

Additional file 2Functional category chart of micro-array results. Percentage of the gene transcripts found differentially abundant in *P. sylvestris *roots after inoculation with *L. bicolor *grouped by functional category. (a) Percentage of genes up-regulated at 1 (□), 5 () and 15 (■) days post inoculation. (b) Percentage of genes down-regulated at 1 (□), 5 () and 15 (■) days post inoculation.Click here for file

Additional file 3 Primers used for the qRT-PCR experiment. List of specific primer pairs used in this study and designed with the following characteristics: primer size 20 bp, Tm 60°C and amplicon size from 50 to 150 bp. Click here for file
