# Supplementary material for: Transcriptional analysis of Pinus sylvestris roots challenged with the ectomycorrhizal fungus Laccaria bicolor
Source: BMC Plant Biol. 2008 Feb 25;8:19. doi: 10.1186/1471-2229-8-19 (PMC2268937; doi:10.1186/1471-2229-8-19)
Supplement: Additional file 1 — Gene transcripts differentially abundant in response to challenge with L. bicolor at 1, 5 and 15 d.p.i. Table showing the 236 ESTs from P. sylvestris found differentially abundant by the mixed model analysis (cut-off fold change was ≥ 1.4 or ≤ -1.4). All the ESTs were divided into 8 patterns, indicated by the numbers 1–8. The transcript ID can be used to retrieve ESTs sequences from the GeneBank or the database at [54]. [file 1471-2229-8-19-S1.pdf]

Functional category	CloneID	annotation	1 dpi M	5 dpi M	15 dpi M	Cluster
**Metabolism**						
	16_G12	inorganic phosphotase	-1,4			1
	22_E07	arginine decarboxylase	-1,6			1
	24_C06	5-methyltetrahydropteroyltriglutamate (methionine synthase)	-1,4			1
	27_D07	enoyl-CoA-hydratase		1,4		4
	38_B04	enolase 2-phosphoglycerate		1,4		4
	NXCI_001_H08	SWP:IFRH_ARATH P52577 ISOFLAVONE REDUCTASE		-1,5		7
	NXCI_018_A08	pectate lyase	1,4			2
	NXCI_032_E01	ASPARTATE CARBAMOYL TRANSFERASE	1,3	1,5		5
	NXCI_040_H05	SWP:METE_MESCR P93263 5-METHYLTETRAHYDROPTEROYLTR		-1,4		7
	NXCI_048_B08	DIHYDROLIPOAMIDE ACETYLTRANSFERASE	1,4			2
	NXCI_055_D02	srg1 protein - aanthocyanidin synthase	1,4			2
	NXCI_066_G08	3-KETOACYL-COA THIOLASE B PEROXISOMAL	-1,4			1
	NXCI_068_C12	polygalacturonase-like protein		1,4		4
	NXCI_082_E07	xyloglucan endo-transglycosylase		2,2		4
	NXCI_094_E12	PECTATE LYASE		1,5		4
	NXCI_098_D10	cytochrome P450	1,4			2
	NXCI_102_C08	PROBABLE MANNITOL DEHYDROGENASE	1,4			2
	NXCI_114_H07	putative uridylate kin	1,6			2
	NXCI_123_C05	ACYL CARRIER PROTEIN, MITOCHONDRIAL PRECUR... 102	1,6			2
	NXCI_127_G06	SWP:SPE1_PEA Q43075 ARGININE DECARBOXYLASE (EC 4.1.1.19)		-1,4		7
	NXNV_002_E08	adenylate kinase		1,4		4
	NXNV_067_G03	nucleotide pyrophosphatase homolog	1,3	1,4		5
	NXNV_074_D01	galactose-1-phosphate uridyltransferase	1,5			2
	NXNV_106_C07	Beta-xylosidase		1,6		4
	NXNV_127_E04	ISOFLAVONE REDUCTASE HOMOLOG	1,3	1,4		5
	NXNV_132_G06	endoglucanase 1 (ec 3.2.1.4) (endo-1,4-beta-glucanase) (cellulase)	1,3	1,4		5
	NXNV_136_H04	EMB:Q43781 Q43781 ASPARTATE AMINOTRANSFERASE (EC 2.6.1.1)		1,7		4
	NXNV_165_G01	methionine synthase		1,4		4
	NXSI_025_H02	alpha-pinene synthase		-1,4		7
	NXSI_040_H09	phosphogluconate dehydrogenase (decarboxylatin... 286 1e-76	-1,4			1
	NXSI_062_B08	no hit		-1,4		7
	NXSI_066_A02	2-oxoglutarate dehydrogenase		1,5		4
	NXSI_083_G03	putative lipase (catalytic hydrolase)		1,7		4
	NXSI_099_F10	adenosylmethionine decarboxylase (EC 4.1.1.50)	-1,7			1
	NXSI_107_B01	Sorbitol dehydrogenase-like protein	-1,4			1
	NXSI_132_B10	LANATOSIDE 15'-O-ACETYLESTERASE PRECURSOR	-1,3	-1,4		7
	NXSI_134_B11	EMB:Q9SFU6 Q9SFU6 PUTATIVE GLUCAN SYNTHASE.		1,4		4

**Energy**						
	01_D05	amino acid selective channel protein		1,4		4
	03_C08	chlorophyll a/b binding protein CP29 precursor		1,4		4
	14_G06	cytochrome C		-1,4		7
	19_B07	23-bisphosphoglycerate independent phosphotase		1,6		4
	21_E04	glyceraldhyde 3-phosph		1,4		4
	22_G10	adenosine kinase-like protein	-1,4			1
	26_D05	ATP synthase c-chain		-1,5		7
	NXCI_002_H04	H+-transporting ATP synthase (EC 3.6.1.34)	1,5			2
	NXCI_008_C01	photosystem ii oxygen-evolving complex protein	1,5			2
	NXCI_018_G04	aldehyde dehydrogenase homolog	1,6			2
	NXCI_026_A11	PUTATIVE GLUTAREDOXIN	1,4			2
	NXCI_031_H08	2,3-BISPHOSPHOGLYCERATE-INDEPENDENT PHOSPHatase		1,6		4
	NXCI_075_C07	PROBABLE NADH-GLUTAMATE SYNTHASE		2,1		4
	NXCI_094_G11	EMB:Q9ZQY2 Q9ZQY2 PYRUVATE DEHYDROGENASE E1 BETA		1,5		4
	NXCI_116_H02	EMB:Q40916 Q40916 NADPH-CYTOCHROME P450 REDUCTASE		-1,5		7
	NXSI_007_H12	SUCROSE SYNTHASE	-1,4			1
	NXSI_060_A06	EMB:Q9ZS54 Q9ZS54 HIGH PI CUZN-SUPEROXIDE DISMUTASE		1,4		4
	NXSI_096_C09	SWP:RUBB_PEA P08927 RUBISCO SUBUNIT BINDING-PROTEIN BETA		1,5		4
	NXSI_116_G04	photoassimilate-responsive protein		1,8		4
**Cell cycle and DNA processing**						
	NXCI_106_H10	CELL DIVISION CONTROL PROTEIN 12 (SEPTIN)	1,5			2
	NXSI_094_B05	EMB:Q43303 Q43303 HISTONE H3 (FRAGMENT		1,6		4
	NXSI_096_C12	no hit			1,4	6
**Transcription**						
	NXCI_008_H10	putative zinc-finger protein	1,4			2
	NXCI_044_A12	PREG-LIKE PROTEIN (FRAGMENT)	1,5			2
	NXNV_002_F08	ELONGATION FACTOR-1 ALPHA	1,4			2
	NXNV_074_G09	putative bZIP transcription factor	1,5			2
	NXSI_021_H06	homeobox protein HAT22		1,4		4
	NXSI_063_D09	PIR:T02995 T02995 unspecific monooxygenase (EC 1.14.14.1) -		-1,7		7

**Protein synthesis**					
	21_F11	elongation factor 1-Al	-1,4		1
	17_H04	acid ribosomal protein		-1,4	7
	22_A01	ribo pro L39		1,6	4
	28_F06	ribosomal protein S3A		1,7	4
	35_B03	40S ribo pro S8		1,6	4
	NXCI_029_D03	40S ribosomal protein S15-like	1,3		2
	NXCI_044_E02	SWP:R13A_PICMA O65055 60S RIBOSOMAL PROTEIN L13A.		1,4	4
	NXCI_050_F08	40S RIBOSOMAL PROTEIN	1,4		2
	NXCI_097_F03	POLY(A)-BINDING PROTEIN	1,4		2
	NXCI_102_G08	60S acidic ribosomal protein	1,5		2
	NXNV_134_B03	PIR:T02039 T02039 acidic ribosomal protein P1a - maize >GEN:2431... -		1,6	4
	NXNV_140_F08	SWP:RS18_ARATH P34788 40S RIBOSOMAL PROTEIN S18.		1,5	4
	NXSI_052_B04	valine--tRNA ligase-like protein	-1,4		1
	NXSI_116_E07	RIBOSOMAL PROTEIN		1,4	4
**Protein fate**					
	02_C01	protease regulatory subunit 7	-1,4		1
	02_C09	antifreeze-like protein (af70) - Norway Spruce		1,6	4
	03_C11	ubiquitin precursor		1,6	4
	03_G03	10 kda chaperonin		1,6	4
	14_G12	polyubiquitin		-1,4	7
	30_B06	ligumine like protease precursor		-1,4	7
	NXCI_032_H03	cullin-like protein	1,4		2
	NXCI_053_D08	EMB:O49977 O49977 UBIQUITIN (FRAGMENT). >GEN:2760349	1,3	-1,4	2
	NXCI_068_D10	COP9 signalosome complex subunit 4		1,4	4
	NXCI_085_H12_F	tau class glutathione S-transferase [Pinus tabuliformis]		1,6	4
	NXNV_123_D06	EMB:Q41067 Q41067 POLYUBIQUITIN. >GEN:1332579		1,5	4
	NXNV_128_D10	Ubiquitin ligase		2,1	4
	NXSI_066_H06	SWP:R11A_LOTJA Q40191 RAS-RELATED PROTEIN RAB11A.		1,4	4
	NXSI_101_H03_F	glycosyl hydrolase family 1 protein [Arabidopsis thaliana]		1,4	4
	NXSI_104_H10	SWP:RERA_ARATH O48670 RER1A PROTEIN (ATRER1A). >PIR:T08570		1,4	4
	NXSI_128_E05	copper chaperone homolog		1,7	4
	NXSI_128_E08	DNAJ PROTEIN HOMOLOG		1,4	4
	NXSI_135_B02	Glycosylation enzyme-like protein		1,9	4

Transport

03_E05	protein translocase	-1,4			1
19_E08	non specific lipid tra	-1,5			1
28_G07	Ran binding protein		1,6		4
33_E11	phosphate transporter [Sesbania rostrata]	-1,4			1
40_A03	Porine MIP1	-1,4			1
NXCI_034_F04	no hit		-1,4		7
NXCI_048_E08	PROBABLE AQUAPORIN	1,4			2
NXCI_070_E11	vacuolar proton pyrophosphatase	-1,4			1
NXCI_102_F06	putative signal sequence receptor	1,6			2
NXCI_124_E12	transporter	1,4			2
NXNV_085_G09	vesicle-associated membrane protein		1,5		4
NXNV_129_F09	VACUOLAR V-H+ATPASE SUBUNIT E	1,4			2
NXSI_116_G10	proton pump putative	1,5			2

Cellular communication and Signal transduction mechanisms

39_C02	putitive casein kinase II catalitic subunit		-1,4		7
02_D01	ser/thre prot kinase	-1,4			1
NXCI_005_G03	PURPLE ACID PHOSPHATASE	1,5	1,6		5
NXCI_067_D08	Ras	1,6			2
NXCI_075_B02	GTP-binding protein		1,7		4
NXNV_010_D03	calreticulin	1,5			2
NXNV_012_H01	TIR/P-loop/LRR [Pinus taeda]	1,3			2
NXNV_077_C07	14-3-3 PROTEIN	1,4			2
NXNV_132_H07	hydroxyproline-rich glycoprotein family protein [Arabidopsis thaliana]		1,5		4
NXNV_136_E11	putative TIR/NBS/LRR disease resistance protein [Pinus taeda]		1,5		4
NXNV_148_H07	CC-NBS-LRR resistance-like protein [Pinus lambertiana]	-1,5			1
NXNV_162_E05	TIR/P-loop/LRR [Pinus taeda]		1,6		4
NXNV_166_H02	EMB:Q17063 Q17063 HEMOLYSIN. >GEN:7144507 U12823 hemolysin	-1,4			1
NXPV_037_F09	NBS/LRR [Pinus taeda]		-1,4		7
NXSI_008_B02	thaumatin-like protein PR5 family [Pinus monticola]		1,5		4
NXSI_021_A09	CLAVATA1 receptor kinase(CLV1)-like protein	1,2	1,4		5
NXSI_026_H06	similar to stress responsive lectin-like cDNAs from rice	-1,4			1
NXSI_036_H01	protein binding / signal transducer	1,4	1,4		5
NXSI_039_G02	putative TIR/NBS/LRR disease resistance protein [Pinus taeda]		1,5		4
NXSI_042_E12	NBS-LRR disease resistance protein homologue [Hordeum vulgare]	1,4			2
NXSI_048_D03	EMB:P92963 P92963 RAB2-LIKE PROTEIN (GTP-BINDING RAB2A		1,6		4
NXSI_052_C04	putative TIR/NBS/LRR disease resistance protein [Pinus taeda]		1,4		4
NXSI_052_D08	putative TIR/NBS/LRR disease resistance protein [Pinus taeda]		1,4		4
NXSI_054_F05	glycine-rich protein homolog [Pinus taeda]	1,3		1,6	3
NXSI_056_B12	no hit		1,4		4

	NXSI_113_D07	no hit		1,4		4
	NXSI_141_G01	Receptor protein kinase-like protein		1,5		4
**Cell rescue and defense**						
	15_F09	multiple stress-responsive zinc-finger protein		1,6		4
	38_A10	metallothionine-like prot EMB 30		1,6		4
	NXCI_002_G06	Blast for putative function	1,5			2
	NXCI_005_C10	LACCASE (EC 1.10.3.2)	1,4			2
	NXCI_022_G01	HEAT SHOCK 70 KDA PROTEIN, MITOCHONDRIAL	1,6			2
	NXCI_045_H07	MLO PROTEIN HOMOLOG 1	1,4			2
	NXCI_055_D03	metallothionein-like protein	1,4			2
	NXCI_076_F07	no hit		2,4		4
	NXCI_094_C09	LACCASE (EC 1.10.3.2)	1,4			2
	NXCI_095_C01	no hit		1,7		4
	NXCI_124_A12	disease resistance protein (TIR-NBS-LRR class), putative [Arabidopsis	-1,4			1
	NXNV_056_F03	PUTATIVE DISEASE RESISTANCE PROTEIN	1,5			2
	NXNV_069_A02	Avr9 elicitor response protein-like		1,6		4
	NXNV_096_C08	PR10 protein [Pinus monticola]			-1,6	8
	NXNV_160_F09	disease resistance protein		1,4		4
	NXNV_162_C02	peroxidase (EC 1.11.1.7) [secretory, cationic]	-1,4			1
	NXSI_002_D03	drought-induced protein		1,7		4
	NXSI_080_D06	PIR:T31428 T31428 fiber annexin - upland cotton >EMB:O82090 O820...		1,7		4
	NXSI_095_H03	no hit		1,5		4
	NXSI_134_G06	PIR:T46629 T46629 lp6 protein - loblolly pine >EMB:Q41083 Q41083...		1,4		4
**Regulation of interaction with environment**						
	23_G07	putative auxin-induced	-1,4			1
	37_E10	gibberellin regulated protein	-1,4	-1,4		7
	37_H11	putative auxin indepen	-1,6			1
	NXCI_018_F09	putative auxin-induced	-1,4			1
	NXPV_038_C08	MtN21 nodulin protein-like	-1,3	-1,7		7
	NXPV_043_G04	MtN21 nodulin protein-like	-1,6			1
	NXSI_091_E03	nifU-like protein		1,4		4
	ST_07_C06	putative auxin-induced		1,5		4
**Development**						
	NXNV_025_F03	PIR:F71433 F71433 probable growth regulator - Arabidopsis thalia... 188		1,4		4
	NXCI_020_A02	embryonic abundant protein	1,4			2
	NXNV_096_G04	argonaute	-1,4			1
	NXNV_129_B12	leaf development protein Argonaute	1,4			2
	NXSI_039_E06	putative floral homeot		1,4		4
	NXSI_041_A07	senescence-associated protein homolog		1,4		4
	NXSI_110_A07	Argonaute (AGO1)-like protein	-1,4			1

**Transposable elements, viral and plasmid proteins**					
	25_C11	RNA-directed DNA polymerase (Reverse transcriptase);	-1,4		1
**Control of cellular organization**					
	02_B01	envelope glycoprotein		1,5	4
	02_F09	histone-like protein	-1,7		1
	06_E01	histone H3	1,4		2
	07_F10	actin depolymerizing protein	-1,6		1
	NXCI_001_A06	beta tubulin [Arabidopsis thaliana]	1,4		2
	NXCI_058_C02	HISTONE H4	1,5		2
	NXSI_048_H03	HISTONE-LIKE PROTEIN	-1,6		1
**Cell, Tissue and Organ differenciation and localization**					
	36_B02	plastid protein	-1,4		1
	NXSI_046_B05	putative endoplasmatic reticulum retrieval protein Rer1B		1,4	4
**Protein with binding function or co-factor requirement**					
	17_A11	DNA binding / RNA binding	-1,4		1
	21_E01	fad binding / aldehyde-lyase/ oxidoreductase/ oxidoreductase,		1,6	4
	37_G12	putative T complex protein 1, theta subunit		1,5	4
	NXCI_101_D04	polyphosphoinositide binding protein Ssh2	1,4		2
	NXNV_005_B04	chloroplast nucleoid DNA binding	1,4		2
**Unclassified proteins and Unknown function**					
	03_F07	no hit	1,5		2
	04_B03	no hits found	-1,4		1
	04_F02	pyruvate carboxylase (EC6.4.1.1)	-1,4		1
	07_A07	hyp pro	-1,4		1
	07_E10	unknown	1,4		2
	12_A02	FS 1 pro		1,6	4
	15_H06	hyp pro		1,5	4
	18_H01	no hit		1,6	4
	25_C10	hypothetical	1,6		2
	27_A08	no hit	-1,5	-1,4	7
	28_F04	no hit	-1,4		1
	30_H03	hyp pro		-1,4	7
	32_F07	hypothetical protein		1,7	4
	34_E10	hyp rpo	-1,5	-1,7	7
	NXCI_009_C07	no hit	1,5		2
	NXCI_027_D03	no hit	1,3	1,4	5
	NXCI_027_E04	no hit		-1,5	7
	NXCI_056_E02	no hit	1,3	1,8	5
	NXCI_071_C03	EMB:Q9LTC1 Q9LTC1 SIMILARITY TO ANKYRIN LIKE PROTEIN.		1,7	4
	NXCI_076_C07	EMB:Q9LVU5 Q9LVU5 SIMILARITY TO RIBOSOMAL PROTEIN L9.		1,4	4
	NXCI_099_A12	no hit		1,5	4

NXCI_121_D01	PIR:T06667 T06667 argininosuccinate synthase (EC		-1,4		7
NXCI_137_B03	no hit	1,4			2
NXNV_003_C10	hypothetical protein		1,4		4
NXNV_044_E12	fiber protein		1,8		4
NXNV_047_B11	hypothetical protein	1,4	1,4		5
NXNV_074_F12	unknown	1,5			2
NXNV_074_G06	no hit	1,5			2
NXNV_083_G05	EMB:Q9NY06 Q9NY06 INTEGRAL MEMBRANE TRANSPORTER	-1,4			1
NXNV_120_E04	EMB:O14597 O14597 NON-FUNCTIONAL FOLATE BINDING		-1,8		7
NXNV_125_D01	no hit		1,4		4
NXNV_125_G04	no hit			1,4	6
NXNV_128_E01	SWP:IF52_NICPL P24922 INITIATION FACTOR 5A-2 (EIF-5A)		1,7		4
NXNV_129_A06	no hit	1,4			2
NXNV_134_A05	PUTATIVE TYPE 1 MEMBRANE PROTEIN		2		4
NXNV_134_H10	no hit	-1,6		1,4	6
NXNV_139_A05	no hit		1,6		4
NXNV_143_B10	no hit		1,7		4
NXSI_001_G04	no hit	-1,5			1
NXSI_005_F10	no hit	-1,4			1
NXSI_021_D01	no hit		1,6		4
NXSI_040_C01	no hit		1,5		4
NXSI_053_D09	no hit		1,4		4
NXSI_079_E12	EMB:Q9LLZ4 Q9LLZ4 PUTATIVE ARABINOGALACTAN PROTEIN.			1,4	6
NXSI_105_A03	no hit		1,7		4
NXSI_107_C02	PIR:T12958 T12958 copper transport protein homolog T6H20.70 - Ar... 67		1,7		4
NXSI_117_C01	no hit		1,7		4
NXSI_134_C12	no hit		1,7		4
