# Supplementary figures and images for: Transcriptional analysis of Pinus sylvestris roots challenged with the ectomycorrhizal fungus Laccaria bicolor
Source: BMC Plant Biol. 2008 Feb 25;8:19. doi: 10.1186/1471-2229-8-19 (PMC2268937; doi:10.1186/1471-2229-8-19)

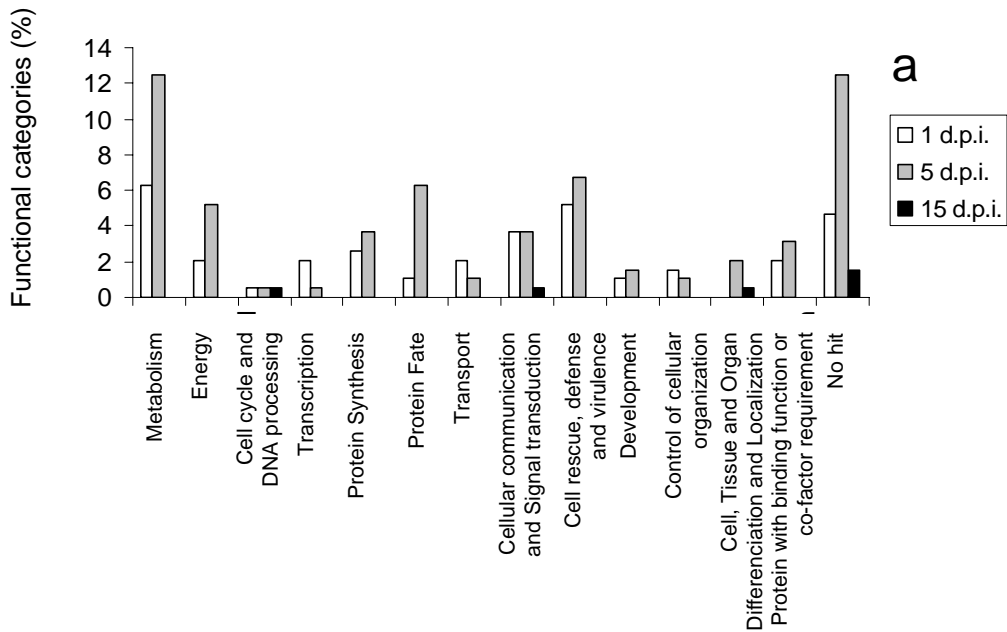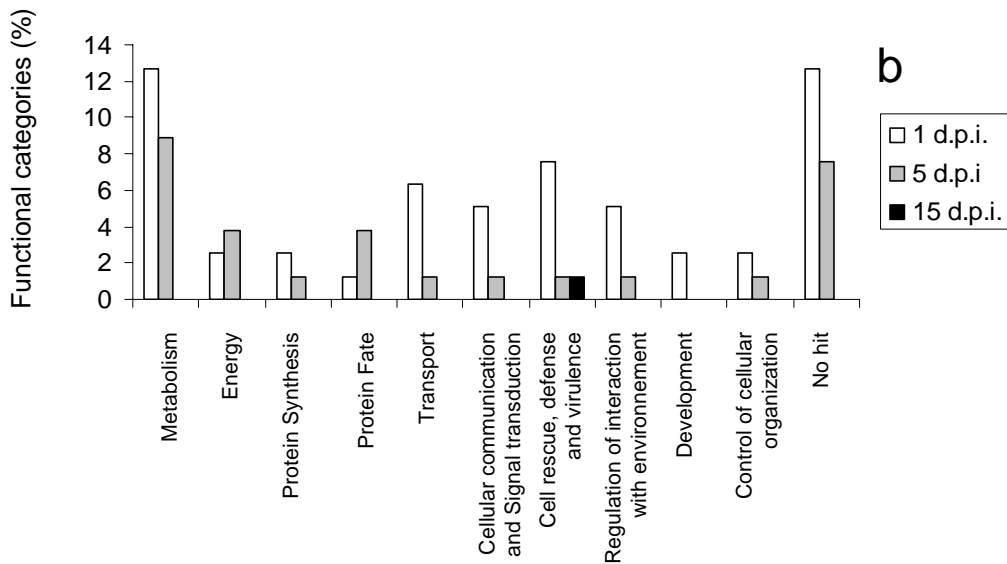

Supplement: Additional file 2 — Functional category chart of micro-array results. Percentage of the gene transcripts found differentially abundant in P. sylvestris roots after inoculation with L. bicolor grouped by functional category. (a) Percentage of genes up-regulated at 1 (□), 5 () and 15 (■) days post inoculation. (b) Percentage of genes down-regulated at 1 (□), 5 () and 15 (■) days post inoculation. [file 1471-2229-8-19-S2.pdf]
