# Supplementary material for: Transcriptional analysis of Pinus sylvestris roots challenged with the ectomycorrhizal fungus Laccaria bicolor
Source: BMC Plant Biol. 2008 Feb 25;8:19. doi: 10.1186/1471-2229-8-19 (PMC2268937; doi:10.1186/1471-2229-8-19)
Supplement: Additional file 3 — Primers used for the qRT-PCR experiment. List of specific primer pairs used in this study and designed with the following characteristics: primer size 20 bp, Tm 60°C and amplicon size from 50 to 150 bp. [file 1471-2229-8-19-S3.pdf]

Table S2: Primers used for the Real Time RT-PCR experiment.

ESTs used for micro-array verification

Gene ID	Putative function	Primer sequence Forward	Reverse
40_A03	membrane intrinsic protein/Porine MIP1	GGCCAGAAAGTTGTCTCTGC	ATGAAGCCCTTCACAACACC
23_G07	putative auxin induced	TGGCTCCTAAGCCCACTAAT	CTCCACCCACTATGAACACG
NXCI_055_D02	anthocyanidine synthase	GATGCGATAAAGGGAGTTGC	CGGAGCACTTTTCTGCTTTT
NXCI_124_A12	disease resistance protein	ACAATTGCCCGAAATTGAAG	ATTGCAACCCACCATTCTCT
NXNV_134_H10	unknown protein 2	AAAGGCATCCCCTGTTTCTT	GGACTAGCCCTGGGACTACC
NXSI_001_G04	unknown protein 3	ATTCATTGCGCTTTTCTGCT	CGCTTTGTGTTTAGCAGGTG
03_F07	unknown protein 4	CGAAGCCATTGCTTTTCTTC	TCCTCATGGATCGAATCTCC
NXCI_018_G04	aldehyde dehydrogenase homolog	CGTGGGGAATTCTCGTTTTA	TTGAGTGCAAGCCTAGCTGA
NXNV_132_G06_F	endoglucanase 1 (ec 3.2.1.4)	AAGTCCGGATTCTTCAGTGC	AGAGTGCTGCAATGACTCCA
NXNV_096_C08_F	PR10/BetV1	AGCGAGCTGGACCTGTAACT	GGGCAAGGCTATCTTCCTTT
NXSI_021_A09	Clavata1	TAATAATCTGAGCGGCAGCA	TTCGAGGAAACATCAAGCTG
NXCI_085_H12_F	tau class glutathione S-transferase	AATACATCGAGGAGGCATGG	TATGGGTCTTCGGGCATAAG
02_C09	subtilisine like protease precursor	CATGAACTATCCGACCATCG	GAGGCGGATCCTATGTTTGT
	kanamycin	GGACGGCGGCTTTGTTG	CTGCGTTGTCGGGAAGATG

ESTs used for gene expression study at 30 d.p.i.

Gene ID	Putative function	Primer sequence Forward	Reverse
23_G07	putative auxin induced	TGGCTCCTAAGCCCACTAAT	CTCCACCCACTATGAACACG
NXSI_021_A09	Clavata1	TAATAATCTGAGCGGCAGCA	TTCGAGGAAACATCAAGCTG
NXCI_055_D02	anthocyanidine synthase	GATGCGATAAAGGGAGTTGC	CGGAGCACTTTTCTGCTTTT
40_A03	membrane intrinsic protein/Porine MIP1	GGCCAGAAAGTTGTCTCTGC	ATGAAGCCCTTCACAACACC
NXCI_085_H12_F	tau class glutathione S-transferase	AATACATCGAGGAGGCATGG	TATGGGTCTTCGGGCATAAG
02_C09	subtilisine like protease precursor	CATGAACTATCCGACCATCG	GAGGCGGATCCTATGTTTGT
NXNV_132_G06	endoglucanase 1 (ec 3.2.1.4)	AAGTCCGGATTCTTCAGTGC	AGAGTGCTGCAATGACTCCA
NXNV_096_C08_F	PR10/BetV1	AGCGAGCTGGACCTGTAACT	GGGCAAGGCTATCTTCCTTT
NXSI_054_F05	glycine-rich protein	GGGGTGAAGGTAAAGTCCAG	GGGCTCATTCAGCTTCTTCT
NXNV_162_h07	Thioredoxin	TCTATCATGGATGCCCAACA	CGCACCATTCTGCAGTAAAA
NXPV_043_G04_F	MtN21 noduline like protein	TCCCACCAGGATAACCAGAC	AGAAGAGAGCCGACAATCCA
NXSI_012_D08_F	Peroxidase	TGAAGGAGTTTGCCCTGAAT	AGCTGGCTCATCTTCACCAT
4_G06	antimicrobial peptide	AATGTGGGTGTTCTAATATCGGCA	AAGCTTTATTTGGGACAACGCC
NXSI_064_A03_F	thaumatin	AATCACAGCGGTTGTAATGG	GAAGGTCGCACATCTCACAC
02_B03	cinnamoyl alcohol dehydrogenase	GTTTGAGGTCGAGGTTGAGG	GTTAATGGGCGAGAGAATGC
NXCI_066_h04_F	acetoacetyl CoA thiolase	TAGGGGTATTGAGGCAAAGG	CATTGCAAACTCCTGCAACT
NXNV_164_H08	xyloglucan endotransglycosylase	TATTCCCCAACAACGAGAGG	TACCCTCATGGGCTGTTTCT
NXCI_098_F10_F	chalcone flavone isomerase	ATGGGTTGCAACTGGAGAAC	TCCAGTGCTTCCTCTTCCTC
34_F04	cinnamoyl CoA reductase	GGAGCAAAAGAGAGGCTGAA	CGGAATAATCCATCAAATCCA
NXSI_012_h05	s adenosylmethionine synthase	GTAAACGGCAGGTTCCAAAA	TGACAATCTCCCAGGTGAAA
